# Exosomal miRNA: Small Molecules, Big Impact in Colorectal Cancer

**DOI:** 10.1155/2019/8585276

**Published:** 2019-10-13

**Authors:** Valentin Vautrot, Gaëtan Chanteloup, Mohammed Elmallah, Marine Cordonnier, François Aubin, Carmen Garrido, Jessica Gobbo

**Affiliations:** ^1^INSERM 1231, Label Ligue National Contre le Cancer and Label d'excellence LipSTIC, Dijon, France; ^2^University of Bourgogne Franche-Comté, EA 318, Besançon, France; ^3^Anti-cancer Center Georges-François Leclerc, Dijon, France; ^4^Faculty of Medicine, University of Burgundy-Franche-Comté, France; ^5^Chemistry Department, Faculty of Science, Helwan University, 11795 Ain Helwan, Cairo, Egypt; ^6^Centre Hospitalier Universitaire, Besançon, France

## Abstract

Colorectal cancer (CRC) is one of the major causes of cancer-related deaths worldwide. Tumor microenvironment (TME) contains many cell types including stromal cells, immune cells, and endothelial cells. The TME modulation explains the heterogeneity of response to therapy observed in patients. In this context, exosomes are emerging as major contributors in cancer biology. Indeed, exosomes are implicated in tumor proliferation, angiogenesis, invasion, and premetastatic niche formation. They contain bioactive molecules such as proteins, lipids, and RNAs. More recently, many studies on exosomes have focused on miRNAs, small noncoding RNA molecules able to influence protein expression. In this review, we describe miRNAs transported by exosomes in the context of CRC and discuss their influence on TME and their potential as circulating biomarkers. This overview underlines emerging roles for exosomal miRNAs in cancer research for the near future.

## 1. Introduction

Colorectal cancer (CRC) is the second leading cause of cancer death in men and the third in women in Europe [1]. 772,000 newly diagnosed cases were registered in 2018, and the estimated number of CRC-related deaths is 242,000. Recently, despite the development of therapies revolutionizing cancer treatment like immune checkpoint inhibitors (e.g., anti-PD-1, anti-PD-L1, or anti-CTLA-4 antibodies), clinical prognosis in CRC remains unsatisfactory, with a 5-year survival rate neighboring 13% at the metastatic stage IV of the disease [2]. An area of study carrying hope for future therapies is the understanding of the relationship between patient prognosis and tumor landscape in primary colorectal tumors. Genetic and epigenetic background of the tumor, as well as tumor microenvironment (TME) composition, are the main factors explaining heterogeneity of response to therapy observed in patients. The TME contains many cell types including stromal cells, immune cells, and endothelial cells. The resulting intra- or intertumoral heterogeneity is of prime importance for all aspects of tumor metabolism and explains the differences in tumor abilities to proliferate, invade, and escape therapy [3–6].

In this context, exosomes are emerging as major contributors in cancer biology. Exosomes are lipid-bilayer, cup-shaped nanovesicles (diameter: ∼50–150 nm) secreted by cells and originating from the endosomal pathway. Exosome release is a common mechanism, and a broad range of cells secrete exosomes, including tumor cells. As a result, exosomes have been detected in a wide variety of biological fluids (e.g., blood, urine, saliva, malignant ascites, and breast milk) [7, 8]. Cumulative evidence suggests that exosomes can establish a fertile environment to support tumor proliferation, angiogenesis, invasion, and premetastatic niche formation. Moreover, they may also facilitate tumor growth and metastasis by inhibiting immune surveillance and by increasing chemoresistance via removal of chemotherapeutic drugs. It has been often reported that tumor cells generate more exosomes than normal cells and that circulating exosome levels are increased in the blood of cancer patients when compared to healthy individuals [9–11]. These features make exosomes interesting reservoirs of potential cancer biomarkers such as proteins, lipids, and RNAs. Although there are some CRC tumor markers used worldwide, there is a particular need for new biomarkers due to technical constraints concerning their detection [12]. In this context, exosomes have become in the last few years an important area of research.

Given their role in TME, exosomes have an essential function in cell-to-cell communication, but they also have specific biological functions. The bioactive cargos received by a recipient cell can modify its physiology by tempering with a vast range of processes [13–17]. Exosomes are implicated in tumor cell proliferation [18], increased migration and invasive properties [19–21], resistance to chemotherapy [22], angiogenesis [23], and escape from the immune system [24]. Although miRNA proportion in exosomes may drastically change depending on the physiological context, tissue, or cell type, they often represent one of the predominant RNAs contained in exosomes [25–27]. Exosomes protect miRNAs from degradation, enabling them to be stably expressed in the extracellular space and to be efficiently integrated by specific recipient cells [28]. Consequently, exosomal miRNAs are also deeply implicated in cancer progression. Therefore, modification or inhibition of exosomal miRNAs might be a potential therapeutic strategy in cancer. In this review, we focus on the impact of miRNA on TME in CRC. First, a description of miRNAs and their biogenesis will be presented, followed by a description of exosome biogenesis and composition. We will conclude by a description of the action of exosomal miRNAs in CRC.

## 2. miRNAs

miRNAs are short single-stranded noncoding RNAs, with a size varying generally between 18 nt and 25 nt (usually 22 nt), that possess the ability to bind complementary target messenger RNAs (mRNAs). miRNAs can induce either translational repression or sometimes degradation of their mRNA targets, thereby constituting a crucial part of posttranscriptional regulation of mRNA expression. Several studies reported the importance of miRNAs in cancer progression, including tumor proliferation, invasion, migration, cell survival, regulation of the immune response, angiogenesis, epithelial-mesenchymal transition (EMT), and cellular stemness [29–35].

In the canonical pathway, miRNAs are at first expressed by the RNA polymerase II as immature stem-loop structure-containing precursors, known as pri-miRNA, of a few hundred to several thousand nucleotides long [36]. However, some pri-miRNAs can be transcribed by RNA polymerase III and some, like miRtrons, are not issued from dedicated transcriptional units but are matured from mRNA introns. A whole cellular machinery is devoted to their processing and nucleocytoplasmic export into functional cytoplasmic miRNAs. First, pri-miRNA precursors are processed into smaller stem-loop pre-miRNAs (approx. 70 nt) by the Microprocessor complex [37]. This complex consists of the Drosha protein, carrying the RNAse activity and DGCR8, that helps determining the proper endonucleolytic cleavage site [38, 39]. Pre-miRNAs are then recognized and exported to the cytoplasm by Exportin-5, where they undergo further endonucleolytic cleavage at the extremities of the stem structure by the RNAse Dicer [40]. The resulting product corresponds to a duplex of 2 complementary miRNAs, the leading strand miRNA or 5p miRNA (formerly at the 5′ extremity of the pre-miRNA) and the passenger strand or 3p or star (∗) miRNA (formerly at the 3′ extremity of the pre-miRNA). This duplex is loaded into a protein complex containing notably Argonaute protein (Ago2), which retains only one of the 2 miRNA strands to form the functionally active RISC complex [41]. The miRNA within RISC complex can recognize and bind to a crucial guide sequence in the target mRNA, located in the vast majority of cases in the 3′-untranslated region (3′-UTR). This sequence, called “seed,” corresponds typically to the position 2 to 8 at the 5′ extremity of the miRNA [42]. Mostly, miRNA pairing with its target is rather imperfect and leads to translational repression or destabilization of the mRNA target [43, 44]. Occasionally, complementarity with the mRNA target is almost total, leading to mRNA cleavage and degradation [45]. As of today, there are around 2,000 entries for human miRNAs in the miRBase database (v22.1) (http://www.mirbase.org/index.shtml). Even if the function of most of them is still unknown, miRNAs are predicted to target most existing mRNAs. Over the years, evidence for their involvement in almost all biological processes accumulated, especially concerning their ability to target oncogenic or tumor suppressor genes in multiple cancer-related cellular pathways [46, 47]. miRNAs are present in significant proportions in blood (and several biological fluids such as saliva, urine, and semen), either incorporated in nucleoprotein complexes with Ago2 protein, nucleophosmin1 protein, within high-density lipoproteins (HDL) particles, or finally encapsulated within exosomes or other extracellular vesicles (EVs) [48–50].

## 3. Exosome Biogenesis and Composition

**Figure 1:**
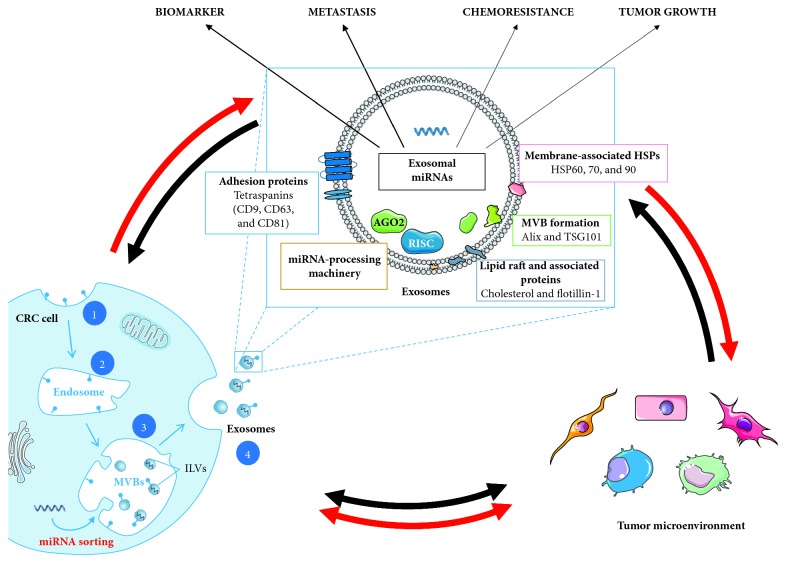
Scheme of exosome biogenesis, composition, and major role in TME modification, in the context of CRC. The biogenesis of exosomes involves 4 different steps: (1) the membrane invagination; (2) endosome formation; (3) generation of the exosome precursors, called intraluminal vesicles (ILVs), by inward budding of endosomes (these accumulations of ILVs are termed as multivesicular bodies (MVBs)); and (4) the fusion of MVBs with the plasma membrane release the ILVs in the extracellular space by exocytosis and become exosomes. Composition: exosomes are composed of different types of enzymes and proteins involved in adhesion, intracellular signaling, immunostimulatory molecules, multivesicular body (MVB) formation, and heat shock proteins (HSPs). Exosomes contain nucleic acids, including miRNA, mRNA, DNA, and small noncoding RNA (snRNA and tRNA). In addition to direct interactions between CRC cells and TME, exosomes, especially exosomal miRNAs, play a key role in the cross talk between cells in TME. CRC cells can release exosomes that will modify TME cells and promote tumor growth, metastasis formation, and chemoresistance. Inversely, stromal cells can also release exosomes that influence tumor cell metabolism. Differential expression of miRNAs within exosomes could also be useful in CRC as biomarker for diagnosis and monitoring.

Exosome biogenesis is initiated by inward membrane invagination of early endosomes to form intracellular multivesicular bodies (MVBs) and then released into the extracellular environment by MVB fusion with the plasma membrane (Figure 1). They differ from other EVs, like ectosomes, which are created by outward budding of the plasma membrane, and apoptotic bodies created during the apoptosis process [51]. Using complex signaling and molecular machineries, like the Endosomal Sorting Complex Required for Transport (ESCRT), newly forming exosomes can incorporate various biologically active molecules. These include different types of nucleic acids and soluble and transmembrane proteins [52, 53]. Among the proteins present in secreted exosomes, some are involved in its biogenesis, like tetraspanins (CD9, CD63, and CD81), Tsg101, and Alix (Figure 1). These proteins are often used as markers, validating exosome enrichment during exosome isolation. Coupled to exosome physical-chemical characteristics (size, density, and buoyancy), they can help discriminating exosomes from other EVs and extracellular particles [54, 55]. Besides, exosome membranes are enriched in lipids (e.g., ceramide, cholesterol, phosphatidylserine, and sphingolipids) and lipid rafts, also playing an important role in their biogenesis and conferring exosomes reinforced rigidity compared to plasma membrane [53]. In particular, ceramide accumulation resulting from conversion of sphingomyelin by sphingomyelinases participates in the formation MVBs [56]. Exosomes also contain proteins that play a functional role in cellular communication, like in antigen presentation. Proteins of the molecular histocompatibility complex (MHC) and various heat shock proteins (Hsp60, Hsp70, and Hsp90) are present in exosomes [57–62]. The incorporation of secreted exosomes into the recipient cell takes place by several mechanisms including macropinocytosis, phagocytosis, endocytosis, or interaction through surface receptors [63, 64].

During their formation, exosomes naturally incorporate cytoplasmic medium. Initially, it was hypothesized to be a nonselective process, resulting in a similar miRNA concentration both in exosomes and parenting cells. Some studies using miRNA for cancer diagnosis or prognosis purposes were implicitly based on the fact that circulating exosomal miRNA levels, especially in body fluids, should reflect accurately the miRNA content of their cells of origin. However, it was rapidly shown in several contexts that the most expressed endogenous miRNAs in tumor or normal cells were not necessarily the ones predominantly secreted into the extracellular environment [65–67]. It is to note, however, that while some miRNA proportions are very different between the cell and the released EVs, this is not always the case. For example, some miRNAs among the most commonly present in both parent cells and exosomes, and that may be potential CRC diagnostic biomarkers present in tissue, plasma, and serum, are miR-192-5p, miR-10a-5p, and miR-191-5p [68, 69].

## 4. Exosomal miRNAs in CRC

The way miRNAs are selectively transported into exosomes for secretion (exosomal sorting) is still not completely clear, although several mechanisms have been proposed [70]. In this section, we will address those hypotheses and the role of different types of biomolecules in miRNAs selective transport into exosomes in the context of CRC.

### 4.1. Role of miRNA Putative Sequence Signals

Several studies suggest the requirement of intrinsic sorting signal sequences in miRNAs, needed for their incorporation into exosomes [71, 72]. One of those sorting mechanisms was described in exosomes from peripheral blood mononuclear cells. It involves recognition of 4-bp RNA motifs, GGAG, by the RNA-binding hnRNPA2B1 protein, provided that it is sufficiently sumoylated [72]. hnRNPC and hnRNPA1, members of the same family of protein, can also bind exosomal miRNAs. Nevertheless, no associated motif has been identified. Another RNA motif, GUUG, was found to be enriched in miRNAs present in exosomes derived from a CRC cell line (SW620) and resembles the GGAG motif recognized by hnRNPA2B1 [73]. This motif was also suggested to be involved in miRNA loading, but it is not known whether it constitutes a specificity of cancer cells or if some RNA-binding proteins, like hnRNPA2B1, intervene in the recognition of this motif.

### 4.2. Role of Exosome Membrane Lipid Composition

It has been reported that the lipid composition of exosome membranes directly influences exosome biogenesis and composition [53, 56, 74]. This also affects miRNA sorting into exosomes. For instance, the level of neutral sphingomyelinase2 (nSMase2), regulating ceramide synthesis, can influence the quantity of miRNA exported through exosomes [70, 75]. In CRC and hepatocellular carcinoma cell lines, it has been shown that sphingomyelin phosphodiesterase 3 (SMPD3), which also generates ceramide from sphingomyelin, is also involved in miRNA encapsulation [76]. SMPD3 inhibition leads to a decrease in exosomal miRNA levels, while the intracellular miRNA level in CRC cells increases. This influence of SMPD3 was, for example, reported for mir-638, a miRNA also downregulated in exosomes of CRC patients which has been proposed as a biomarker [77, 78].

### 4.3. Role of Proteins Involved in miRNA Biogenesis and Functions

The miRNA maturation process is connected with miRNA export in exosomes and endosomal trafficking. Knockout of Ago2 leads to the selective decrease of certain miRNA populations in exosomes from several cell lines [79]. In addition, components of the RISC complex can colocalize with MVBs, when MVBs turnover into lysosomes is blocked [80]. In exosomes derived from different cancer cell types, all the essential elements required for pre-miRNAs processing into mature miRNAs, including Dicer and Ago2, are available [10, 81]. When transfected with *C. elegans* pre-miRNA, those exosomes were able to process this pre-miRNA into mature miRNA. This was confirmed to be a Dicer-dependent process. In contrast, miRNA maturation machinery was not detected in exosomes from nontumorigenic cancer cells. CD43, a suspected mediator of active protein transported into exosomes, is enriched in those exosomes. This protein is responsible for the increased level of Dicer, further linking exosome processing with miRNA biogenesis [82, 83]. Probably also related to miRNA biogenesis, it was observed that passenger-strand (3p) miRNAs seem predominant in CRC cell-derived EVs compared to their 5p counterparts [84].

One mechanism highlighted in CRC cells underlines a possible role of the small GTPase KRAS in miRNA sorting. KRAS mutations occur in more than a third of sporadic colorectal cancers, and it has been associated with several other cancers, in particular, regarding tumor aggressiveness [85–87]. Exosomes secreted by KRAS mutant CRC cells can induce growth and migration of wild type (WT) cells [88, 89]. KRAS mutations can influence the recruitment of Ago2, involved in miRNA maturation and secretion, into the nascent exosome [90, 91]. In particular, KRAS mutations affect exosomal encapsulation of several miRNAs implicated in CRC, such as the oncogenic miR-10b, which is selectively retained in WT KRAS-cell exosomes [90]. A higher rate of tumor-suppressor miRNA sequestration and decreased level of oncomiRs were observed in exosomes compared to their parent CRC cells [92]. This process seems to depend on the major vault protein (MVP), a proposed miRNA-binding protein responsible for sorting miRNA to exosomes that is overexpressed in multidrug-resistant cancer cells [93, 94]. Since tumor cells can selectively retain oncomiRs, it was suggested as a phenomenon favoring tumor growth and progression [90, 92]. Moreover, exosome secretion could be used as a way to discard tumor-suppressor miRNAs or other molecules that promote apoptosis, cell cycle arrest, or differentiation, thus also enhancing tumor cell growth and metastasis. This selective secretion was, for example, observed for several tumor-suppressor miRNAs, like miR-23b, miR-224, and miR-921 [95]. In that study, it was shown to be dependent on an important exosome transporter, Rab27, and to significantly affect metastasis and angiogenesis potential of bladder carcinoma cell lines. Because most studies rather focused on how the miRNAs secreted from tumor cells influence their environment, these interesting data need further investigation.

As we will see in the following sections, there are hints that these mechanisms can be disturbed during the tumorigenic process in CRC, explaining the differences systematically observed in miRNA content between exosomes from healthy individuals and CRC patients.

## 5. Exosomal miRNAs Influence CRC Tumor Microenvironment

Exosomal miRNAs in the tumor microenvironment (TME) have a significant influence on tumor development and progression but are also able to transfer the ability to resist to the anticancer therapy [96–98]. The following section will present the main exosomal miRNAs (exomiRs) proven to be functionally implicated in CRC tumor metabolism. These include miR-21, the miR-200 family, the miR 17∼92 cluster, and miR-1246 alongside other relevant miRNAs. Information on expression, role as a biomarker, and function of each miRNA in CRC will be further detailed in the following sections. Available data are summarized in Table 1.

### 5.1. Exosomal miR-21 and miR-155

#### 5.1.1. Expression and Role as Biomarker

miR-21 was the first shown to be expressed at high levels in the exosomes of 3 different CRC cell lines (HCT-15, SW480, and WiDr) [99]. Interestingly, these tumor-derived exosomes were found to be transferred to normal hepatic and lung cell types, preferred metastasis targets for colon tumors. Later, it was confirmed that miR-21 was overexpressed not only in colon tumor tissue and in liver metastases tissue, but also in plasma exosomes of CRC patients [11, 100]. Exosomal miR-21 expression in plasma has been significantly correlated to its expression on tumor tissue, but also to disease stage, occurrence of liver metastasis, and prognosis. Other studies have reported that this exomiR can be used as a biomarker in CRC [101] but also as a general biomarker of gastrointestinal cancers including esophagus, rectum, and pancreas [102].

miR-21 was systematically found in miRNA populations characterizing circulating exosomes from plasma, feces, and serum in the context of colorectal cancer, as well as in exosomes from different CRC cell lines [84]. It is thereby possible that the circulating biomarker value of miR-21 comes mostly from its presence in exosomes. Nevertheless, it was recently shown that nonvesicular Ago2-associated miR-21 was actively released from HT29 CRC cell lines and that its levels could surpass those of EV-encapsulated miRNA in the absence of chemical lysis [120].

#### 5.1.2. Function in CRC

**Figure 2:**
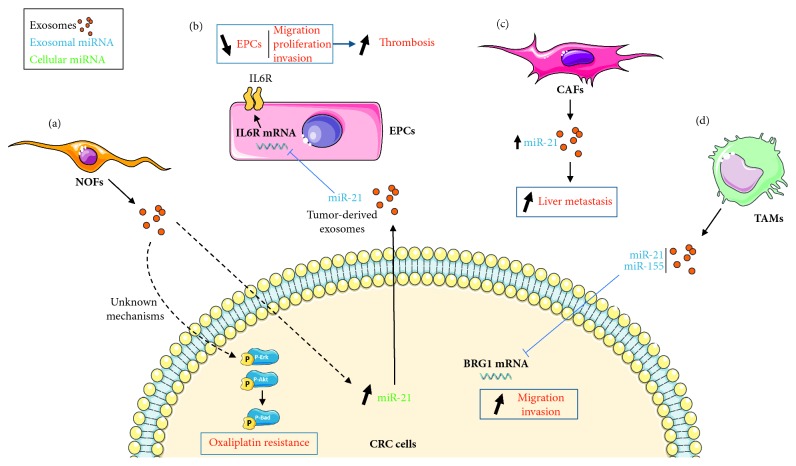
Proposed models for the role of exosomal miR-21 in CRC development. (a) Fibroblast-derived exosomes have an effect on CRC cells. The internalization of normal fibroblast- (NOF-) derived exosomes into CRC cells leads to an increase of cellular miR-21 and to the activation of phospho-Erk/Akt pathway, leading to oxaliplatin resistance. (b) CRC cells release miR-21-containing exosomes that are able to inhibit endothelial progenitor cell (EPC) IL6R mRNA transcription, leading to a reduced migration, proliferation, and invasion and favoring thrombosis in CRC. (c) Cancer-associated fibroblasts (CAFs) secrete miR-21-overexpressing exosomes which increase liver metastases. Tumor-associated macrophages (TAMs) also release miR-21-containing exosomes that can negatively regulate BRG1 mRNA in CRC cells and lead to an increased migration and proliferation.

Exosomal signal of stromal origin, such as exosomes produced by normal fibroblasts (NOFs), can be transferred to CRC cell lines (DLD1 or SW40) and lead to an increased expression of miR-21-5p. This transfer also leads to increased phosphorylation of cell-signaling factors Erk, Akt, and Bad, resulting in an increased resistance to the anticancer drug oxaliplatin (Figure 2(a)).

Overexpression of mir-21 observed in exosomes from CRC tissues leads to a drastic reduction of endothelial progenitors cell (EPC) migration, proliferation, and invasion properties [121]. EPCs are circulating progenitor cells of different types, able to differentiate into functional endothelial cells and to participate in new vessel formation and blood vessel regeneration. This effect on EPCs occurs most likely through direct targeting of interleukin 6 receptor (IL6R) (Figure 2(b)). Since EPCs promote thrombus repair and resolution, it was hypothesized that it led to a higher incidence of deep-vein thrombosis, a prognostic factor in cancer patients.

In the context of CRC, stromal cells themselves can also release miR-21 into the TME, in agreement with previous observations based on stromal microdissections [122]. The altered cancer-associated fibroblasts (CAFs) produce miR-21 rich exosomes, both in regards to intracellular levels but also to the exosome content of NOFs. This increased expression in exosomes is associated with an increase in liver metastasis. These data were confirmed *in vivo* in mouse orthotopic xenografts (Figure 2(c)) [103].

miR-21 is enriched in exosomes produced by M2 macrophages, as is the oncomiR miR-155 [104]. M2 macrophages serves as *in cellulo* model for tumor-associated macrophages (TAMs) present in the TME. These macrophages promote proliferation, invasion, and metastasis of cancer cells, angiogenesis, and immune escape [123]. In CRC cells, both miR-21 and miR-155 are able to target the transcriptional regulator BRG1, resulting in increased migration and invasive behavior (Figure 2(d)). Exosomal miR-21 and miR-155 were thus suggested to be partly responsible for TAM's effects on CRC cells.

### 5.2. Exosomal miR 17∼92 and 25∼106b Clusters

#### 5.2.1. Expression and Role as Biomarker

Members of the 17∼92 miRNA cluster (miR-17, -18a, -19a, -19b-1, -20a, and -92a-1) were detected in high proportions in exosomes from the LIM1863 CRC cell line, alongside the members of its paralog cluster miR 106b∼25 (mir-25b, -93, and -106b) [84]. Interestingly, miR-17, -19a, -20, and -92a are specifically enriched in exosomes as compared to several of their parent CRC cell lines, indicating their potential importance in exosomal communication [106]. miR-19a, -19b, and -92a are also upregulated in serum exosomes of CRC patients compared to those of healthy individuals, which has been linked to liver metastasis recurrence [9]. miR-19a-5p, in particular, was also suggested as a convincing biomarker for CRC severity and lymph node metastasis appearance and prognosis. The upregulation and biomarker value for disease stage of miR-19a-5p, as well as miR-19a-3p and miR-17-5p, were confirmed in serum exosomes of CRC patients [101, 106]. Moreover, miR-17-5p, -18a-5p, -19a/b-3p, -20a-5p, and -92a-1-5p expression is significantly upregulated in exosomes derived from metastatic CRC cell line SW620 compared to exosomes derived from the nonmetastatic SW480 cell line [107].

#### 5.2.2. Function in CRC (miR-25-3p)

Concerning the precise role of those two miRNA clusters in exosomal regulation, the main set of evidence comes from miR-25-3p and its action on the formation of premetastatic niche. Serum-derived exosomal miR-25-3p expression has been associated with higher rate of metastases in CRC patients [23]. *In vitro* data suggest that CRC cell-derived exosomes containing miR-25-3p can enter endothelial cells and induce migration, angiogenesis, and vascular permeability. This was confirmed *in vivo* by tail vein injection of exosomes in mice, leading to a higher rate of metastases formation in liver and lungs, in a miR-25-3p-dependent manner. It was suggested to result from miR-25-3p targeting of the transcription factor KLF2. KLF2 negatively regulates expression of angiogenesis factor VEGFR2 and of KLF4, a transcription factor regulating the integrity of endothelial barrier and tight junctions.

### 5.3. Exosomal miR-200 Family

#### 5.3.1. Expression and Role as Biomarker

Another important family of exosomal miRNAs in CRC is the miR-200 family, which encompasses two miRNA clusters. The first regroups miR-200a, -200b, and miR-429, and the second regroups miR-141 and 200c. Lower expression levels of miR-200c and miR-141 were significantly associated with better survival, in both the tumor draining vein (mesenteric) plasma and the corresponding exosomal fraction [124].

#### 5.3.2. Function in CRC

**Figure 3:**
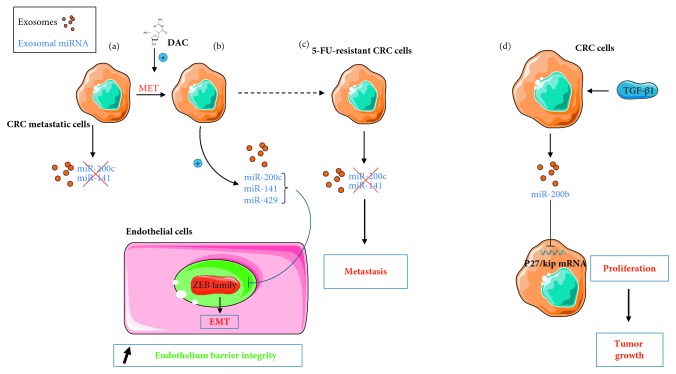
Proposed models for the dual roles of exosomal miR-200 family members on TME in CRC. (a) Upon decitabine (DAC) treatment, CRC cells enter a MET process that stimulates the release of miR-141/miR-200c enriched exosomes. (b) In endothelial cells, exosomal miR-200c, -141, and -429 can also inhibit the expression of transcription factors belonging to the ZEB family, activators of EMT. (c) On the contrary, 5-FU-resistant CRC cells release exosomes without miR-200 family members, favoring EMT. (d) CRC cells exposed to TGF-*β*1 release miR-200b-enriched exosomes that inhibit p27/kip mRNA, leading to an increased proliferation.

On one hand, miR-141, -200c, and -429 have a protective effect against tumor progression, but only seemingly active in absence of the epithelial-mesenchymal transition (EMT), a crucial feature of cancer cells acquiring metastatic properties. Indeed, CRC metastatic cells (SW640) treated with the anticancer drug decitabine (DAC) reacquire epithelial characteristics by undergoing EMT reversal (MET). This includes inhibition of their migration and invasion properties. During this phenomenon, exosomal miR-141 and -200c expression increases, while remaining unaffected when DAC has no effect on EMT, like in the corresponding primary tumor cell line (SW480) [108]. This suggests that miR-141 and -200c expression in exosomes is negatively impacted by EMT and positively impacted by the mesenchymal-epithelial transition (MET) (Figure 3(a)).

miR-200c and also miR-141 and miR-429 are expressed in exosomes of naïve CCL27 CRC cell spheroids in 3D culture models [109, 110]. In cells surrounding the tumor, they directly target several members of the ZEB family, which are transcription factors involved in EMT (Figure 3(b)). As a result, miR-200c inhibits EMT in the lymphatic endothelial cells (LECs) co-cultured with CRC spheroids [110], and miR-200c, -141, and -429 inhibit EMT in co-cultured blood endothelial cells (BECs) [109]. Since exosomal expression of those miRNAs is lost in 5-FU (5-fluorouracil) chemoresistant spheroid cultures, surrounding cells engage in EMT transition, visibly weakening the lymphatic (LEC) and blood (BEC) endothelial barriers. By facilitating the crossing of those barriers by CRC cells, this phenomenon could explain increased metastasis occurrence in chemoresistant CRC. Thus, the data suggest that transfer of those miRNAs through exosomes contribute to preventing cell permeation into epithelia and maintaining tissue and organ integrity in normal physiological cell conditions (Figure 3(c)).

On the other hand, an oncogenic effect of exosomal miR-200b derived from CRC cells (HCT-116 and SW480) was also reported [98]. Exosomal miR-200b level is increased in cells treated with TGF-*β*1 in a dose-dependent manner and is responsible for the proliferative properties of the resulting exosomes, observed on another CRC cell line. These results were assigned to direct targeting of the antiproliferative cyclin-dependent kinase inhibitor 1B (p27/kip) by miR-200b (Figure 3(d)). The decrease of p27/kip expression was confirmed *in vivo* following miR-200b injection in xenograft of tumor cells. This also led to an increase in tumor size, as expected.

### 5.4. Exosomal miR-1246

#### 5.4.1. Expression and Role as Biomarker

It was reported that miR-1246 is specifically upregulated in exosomes derived from several CRC cell lines and carcinoma cell lines from the cervix (HeLa), bladder (T24), prostate (PC-3), and liver (HepG2) [11, 23]. In a meta-analysis encompassing literature data from blood, urine, and other bodily fluids, it was the best performing miRNA biomarker for gastrointestinal cancers in terms of specificity and sensitivity [102]. This was in agreement with a previous high-throughput experimental study in serum exosomes, concluding that miR-1246 was the best potential miRNA biomarker for CRC diagnosis in serum together with miR-23a [11].

#### 5.4.2. Function in CRC

**Figure 4:**
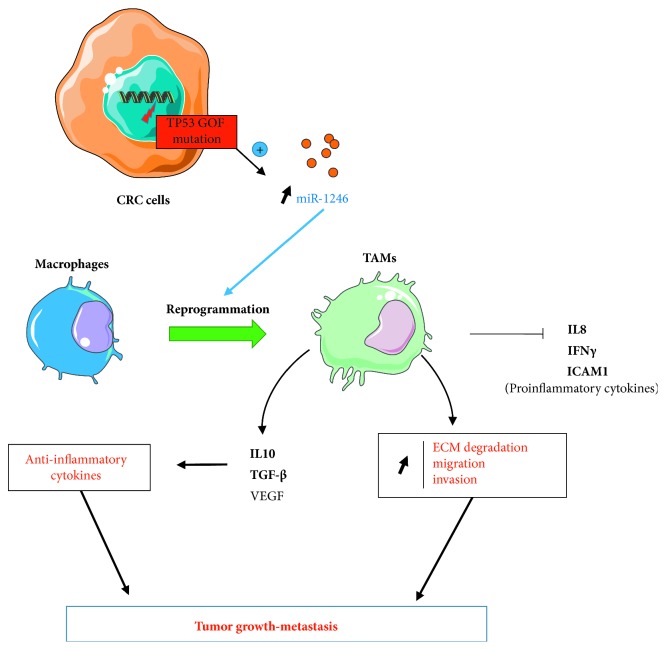
Proposed models for the effects of exosomal miR-1246 on the TME. p53 mutations resulting in gain of function (GOF) in CRC cells induce the release miR-1246-overexpressing exosomes. Exosomal miR-1246 can induce a switch of macrophage phenotype towards a tumor-associated phenotype (TAM), modifying tumor inflammatory state. It leads to a decreased secretion of proinflammatory and increased secretion of anti-inflammatory cytokines. TAMs also present enhanced ECM degradation, migration, and invasion properties. Exosomes are represented as small orange circles.

Through its action on inflammation, exosomal miR-1246 holds an important role in TME. It was shown that this action was linked to the presence of p53 (*TP53*) mutations in CRC cells. These alterations are one of the most frequent genetic traits of human cancers [111, 125]. The experimental proofs obtained both *in vitro* and *in vivo* allowed to establish a model, in which the presence of *TP53* mutations in CRC cells, specifically resulting in a gain of function (mutp53), led to an increase of miR-1246 levels in exosomes [111]. Exosomal miR-1246 can induce reprogramming of macrophages towards a TAM phenotype, a hallmark of solid tumors associated with poor prognosis (Figure 4). Indeed, those mutp53-reprogrammed TAMs possess an anti-inflammatory cytokine secretion signature (e.g., IL-10, TGF-*β*, or VEGF). Moreover, their proinflammatory cytokine secretion (e.g., IL-8, IFN-*γ*, and ICAM-1) is decreased. Mutp53-reprogrammed macrophages also revealed a marked stimulation of extracellular matrix (ECM) degradation activity and enhanced migration and invasion properties. As a consequence, the anti-inflammatory, immunosuppressive, promigration, and proinvasion properties obtained by such macrophages promote tumor growth and metastatic burden to liver and lungs, as confirmed in mice hetero- and orthotopic xenograft models.

Interestingly, a pull-down experiment revealed an association of miR-1246 with hnRNPA2B1, which is suggested to be responsible for miRNA sorting in exosomes in its sumoylated form [72, 111]. The motif recognized by hnRNPA2B1 (GGAG) is carried by miR-1246, and hnRNPA2B1 sumoylation is 3 times higher in mutp53 CRC cells than in the WT CRC cells, suggesting that changes in this mechanism are involved in exosomal miR-1246 oncogenic properties.

### 5.5. miR-149 and miR-96-5p, -486-5p, -6869-5p, -8073, and -193a: Tumor Suppressors

GPC1 (glypican-1) is a member of the heparan sulphate proteo-glycan family and is an important biomarker, found in several types of cancer (breast, pancreatic, and glioma) and involved in angiogenesis and tumor growth [126–129]. It was shown that GPC1 overexpression in CRC cells induces EMT and promotes cell invasion and migration [112]. The miR-149 gene is located within an intron in the *GPC1* gene. miR-149 and miR-96-5p are both able to directly target GPC1 mRNA, resulting in proapoptotic and antiproliferative effects in CRC cells *in vitro* and *in vivo*. Both miRNAs are downregulated in exosomes from CRC tissues or in plasma when compared to those of healthy individuals, while the exosomal GPC1 level is increased [113].

It was shown that exosomal miR-486 was upregulated within plasma exosomes and whole plasma of CRC patients. Therefore, it was suggested as a CRC diagnosis biomarker [114]. However, miR-486-5p possesses a tumor suppressor activity via inhibition of cell proliferation. Indeed, it targets directly PLAGL2, a transcription factor for *β*-catenin and IGF2 that promotes both proliferation and metastasis and inhibits apoptosis. Nevertheless, in CRC tissues, it has been shown that miR-486-5p expression is inhibited due to a high rate of DNA methylation of its promoter region. The consequent upregulation of PLAGL2/*β*-catenin/IGF2 pathway leading to proliferation and migration was confirmed in CRC cells.

miR-6869-5p downregulation was also observed in tumor tissues and serum exosomal fractions from CRC patients, and it was proposed as a potential biomarker of CRC prognosis [115]. The tumor-suppressor activity of miR-6869-5p was supported by direct targeting of TLR4, subsequently inhibiting TNF‐*α* and IL-6 production in CRC cells via the TLR4/NF-*κ*B signaling pathway, leading to a decrease in cellular proliferation.

While there is no difference between intra- or extracellular miR-8073 in normal colorectal cells, it is at least 60 times more expressed in exosomes from CRC cells than in the intracellular extracts. Mizoguchi et al. demonstrated that it can directly target several factors involved in survival, proliferation, and antiapoptosis (FOXM1, MBD3, CCND1, KLK10, and CASP2), resulting in its antiproliferative properties *in vitro* and its effect on tumor growth *in vivo* [116].

Finally, miR-193a was shown to have a tumor-suppressor activity by targeting Caprin1, an upstream activator of the G1/S-specific cyclin-D2 (Ccnd2) and the proto-oncogen transcription factor c-Myc [92]. This causes G1 cell cycle arrest, leading to inhibition of cell proliferation. miR-193a is present at high levels in the exosomal fraction of CRC patients' serum, particularly in advanced stages, with high risks of metastasis. It was also demonstrated that miR-193a sorting into exosomes, which is increased in CRC, was caused by the MVP transporter [92].

### 5.6. miR-10b: Indirect Oncogenic Activity via CAF Transformation

miR-10b was detected in exosomes derived from multiple types of cancer cells and was particularly enriched in exosomes from CRC cells [90], but also breast cancer [130] and non-small cell lung cancer [131]. It can target directly PIK3CA, thus inhibiting PI3K/Akt/mTor pathway activity, closely associated with the inhibition of cell migration and invasion [117, 132]. Moreover, exosomes derived from CRC cells that contain miR-10b can be transferred to fibroblasts. In the target cells, this results in increased expression of TGF-*β* and SM α-actin. Expression of those genes are characteristics of myofibroblast-like CAF phenotype [103], and should stimulate tumor cells proliferation. miR-10b was shown to be particularly sensitive to mutation in the exosomal sorting protein KRAS, as KRAS mutations lead to a decreased incorporation of miR-10b in secreted exosomes [90].

### 5.7. miR-142-3p and 196b-5p: Stemness-Inducing miRNAs

Bone marrow-derived progenitors are an additional important type of stromal cells present in tumors, which are able to release cytokines and exosomes and influence TME. Bone marrow mesenchymal stem cells (BM-MSCs) are indeed able to release exosomes that increase markers of stemness (Oct4, lin28, KLF, Bmi-1, CD44, and SOX2) in CRC cells and their subsequent invasion, adhesion, and drug resistance properties [118]. It has been shown that this effect relies on the influence of miR-142-3p, present in exosomes, which can directly target Numb, an inhibitor of the Notch stem cells pathway [133]. Consequently, exposure to miR-142-3p-containing BM-MSC exosomes results in a boost of tumorigenesis and tumor metastasis, but not tumor weight and size, as shown in orthotopic grafts in mice [118].

miR-196b-5p influences stemness by targeting directly SOCS1 and SOCS3, modulators of stemness pathways, resulting notably in increased STAT3 transcription factor activity in CRC cells and tissues. It increases the production of antiapoptotic factors, like Bcl-2, Bcl-xL, and BIRC, and stem cell factor markers, like NANOG, Bmi-1, OCT4, and SOX2, and increases resistance to the drug 5-fluorouracil [119]. miR-196b-5p was confirmed to be dramatically upregulated in serum exosomes of CRC patients, in a much more distinct manner than in the whole serum, and associated with poor prognosis.

### 5.8. Lead on miR-210 Importance in EMT

It has been observed that a subpopulation of HCT-8 CRC cells became nonadherent after a few days of culture. Additionally, their proportion increased with culture time, and they developed chemoresistance properties by undergoing EMT [19, 134]. This metastatic-like phenotype can be spontaneously reverted in new cell-free cultures, but not in the presence of other HCT-8 cultured cells. Indeed, the reverse MET phenomenon was inhibited by exosomes produced by cultured cells. As miR-210 is significantly upregulated in HCT-8 exosomes, the authors suggested that it may play a role in MET inhibition [19].

### 5.9. Other miRNAs

Finally, additional miRNAs found in exosomes were also identified as potential biomarkers in CRC patients. Even if they are not described to play a role in exosomes mode of action, we tried to make a list as exhaustive as possible of the main reported ones in the current state of the art. Data are outlined in Table 2 [105, 135–139].

## 6. Concluding Remarks

Cell-to-cell transfer of miRNAs by means of exosomes, released by both stromal and tumor cells, plays an important role in tumor progression and metastasis. Several technical obstacles should be overcome to allow improved exosome characterization and further research in particular subjects that remain less covered. Study of miRNA targets and role in CRC provides great hopes for better understanding and characterization of tumor properties, diagnosis and personalized medicine, and innovative therapeutic approaches. These aspects will be briefly discussed in the following sections.

### 6.1. Limitations regarding Exosome Isolation Methods and Exosome Purity

Exosomes constitute great reservoir of biomarkers since they preserve miRNAs from extracellular environment and have dedicated roles and a specific biology. For example, in one of the first high-throughput characterization of exosomal miRNAs in CRC cells by Ji et al., almost a third of miRNAs from a subpopulation of exosomes were not reported as implicated in colon cancer before [84]. However, exosomal miRNA studies are limited due to a few technical issues. It is currently almost impossible to achieve a very high degree of purity without sacrificing yield when isolating exosomes. There are many approaches to isolate exosomes from the same medium, which are fundamentally different in their principles, resulting in different yields and degree of purity and often used according to the objectives of downstream applications [140].

Unfortunately, it has been shown that the purification method has a great impact on the exosome population obtained, including on miRNA content [141, 142]. To help ensure that the obtained isolates are enriched in exosomes, several validation tests also have been proposed. These tests typically include nanoparticle tracking analysis (NTA), exosomes markers detection by western blot, or examination of exosomes by electron microscopy. Studies on the research of biomarkers, mentioned along this review, used different methods of purification, as summarized briefly in Tables 1 and 2. Both the purification method and validation of exosomal enrichment experiments have to be taken into account during result interpretation.

### 6.2. Nonexosomal vs. Exosomal Extracellular RNAs

Concerning the vesicle-free part of circulating miRNAs secreted by cells by other means, the involved mechanisms are still unclear. Their release could also largely result from cellular lysis. Compared to miRNAs contained in EVs, it is not clear if their role in tumor transformation and progression is negligible or not. To elucidate these roles entirely will remain difficult due to current technical impediments limiting the purity of isolated exosomes and EVs in general [53, 140]. This state of the art was recently backed up by a study in rat serum and plasma, showing that vesicle-free miRNAs are also present in EV fractions after isolation. Moreover, even Ago2-associated part of vesicle-free circulating miRNAs could result from either cellular or exosomal lysis [10, 143].

If the proportion of circulating miRNAs present in exosomes compared to free circulating miRNAs remains elusive, it seems that only a small fraction (down to 10%) of plasma miRNAs are vesicular, whereas in serum or saliva, the majority of miRNA are concentrated in exosomes [48, 49, 144, 145]. In plasma, the fraction of miRNAs present in the vesicle fraction is strongly dependent on the identity of the miRNA considered. Some, like let-7a, were found predominantly enriched in vesicle fractions compared to vesicle-free fractions, while others like miR-16 and miR-92a are preferably associated with circulating Ago2 and seemingly absent from vesicles under physiological conditions [48]. However, it is worth noting that at least in one report (in high-risk colorectal adenomas), 2 serum exosomal miRNAs were considered less efficient biomarkers than their whole serum miRNA counterparts despite their correlated expressions [146]. Although it seems that isolated vesicle-incorporated miRNAs are more stably expressed and constitute more reliable cancer biomarkers than their vesicle-free counterparts [28, 147].

### 6.3. Exosome Subpopulations in CRC and Their Advantages

Different CRC cell types produce different populations of exosomes. For example, it was shown that CRC cell line exosomes do not contain the same combination of tetraspanin proteins, exosomal markers involved in exosome biogenesis [99]. On the same note, Chen et al. showed that miRNA composition of SW480- and SW620-derived exosomes is significantly different, with more than a third of the miRNAs detected being differentially expressed between the 2 types of exosomes [107]. Moreover, while miRNAs are sorted into exosomes in a differentiation state and cell-type dependent fashion, several types of exosome populations with diverse morphologies have been reported to be secreted by the same cells, in particular in colorectal cancer [148, 149]. The LIM1863 CRC cell line can produce two mutually distinct populations of exosomes, one presenting A33 and the other EpCAM surface proteins, an important cancer-initiating marker in CRC and pancreatic cancer [150, 151]. Their protein and miRNA populations vary significantly between each exosome type and previously determined proteomes of other exosomes, suggesting different effects and/or target recipient cells [84, 150]. Indeed, it was shown that exosomes are tailored to target specific types of recipient cells [152, 153]. This could provide an explanation for the site-specific formation of metastases in colorectal cancer, e.g., the liver, lungs, and lymph nodes. Moreover, their compositions reflect not only this tailoring, but also regulatory events arising in the secreting cell [80]. Exosomes could thus give great advantages for both study of TME and discovery of biomarkers. Indeed, a given exosome population could thereby directly inform us about particular cell types and events they were exposed to, with great specificity. These subpopulations may contain multiple determinants of tumor metastatic potential. The complex interplay between exosome subpopulations, their specific contents, and their potential target cells needs further investigation.

### 6.4. A Word on Therapeutic Perspectives

Studies on exosomal miRNAs may soon be applied to the clinical setting, as new therapeutic approaches using delivery of miRNA mimics or miRNA antagonist on tumor sites are in development. Several clinical trials concerning the use of miRNAs in the treatment of CRC are currently ongoing and gain more and more interest from biopharmaceutical companies [154]. Furthermore, exosomes themselves constitute a great strategy for the delivery of those new therapeutic actors. Freely circulating miRNAs are rather instable in blood [48] and are also negatively charged, rendering delivery through cell membranes difficult even *in vitro*. Exosomes, on top of their low immunogenicity and cytotoxicity, may enhance therapy deliverability and protect molecules from RNAse activity, making them suitable therapeutic vectors for CRC treatment [155, 156]. Treatment of cells with FF/CAP18 (analog of cathelicidin LL-37), a peptide limiting cancer cell proliferation, induces production of exosomes with antiproliferative properties [157]. This effect is suspected to come from the expression of exosomal miRNAs miR-584-5p, -1202, and -3162-5p. Kyuno et al., have also shown that it is possible to tailor exosomes for therapeutic purposes by transfection with tumor-suppressor miRNAs [158]. The miRNAs in question were miR-342 and -498, which target Claudin7 (cld7) and EpCAM, respectively. Coupled with exosomal expression of ectopic Tspan8, shown to enhance internalization by cancer cells [152], it was sufficient to inhibit tumor growth, motility, and invasion, especially by affecting stemness traits.

To conclude, exosome-carrying miRNAs originating both from the stromal and the tumor cells have a major role in CRC TME. Exosome encapsulation enables miRNA expression in extracellular space and involvement in cell-to-cell communication. Therefore, miRNAs can influence cell inflammatory environment, differentiation status, proliferation, survival, migration, invasion, and angiogenesis properties. Being stably released in the circulatory system, it has been shown at least through venal injection that they can influence distant cell barrier permeability, underlining the role they play in premetastatic niche formation. The delivery of exosome cargo into specific types of target cells may also be one of the mechanisms explaining organ preference of cancer metastasis. For all those reasons, exomiRs constitute a key target for the discovery of biomarkers and new therapeutic approaches in CRC, and an important axis of research in the near future.

## Figures and Tables

**Table 1:** Summary of exosomal miRNA with known functions in CRC. The main source of exosomes in the publications is indicated. Exosomes isolation techniques employed in the literature are indicated with their respective exosome enrichment validation procedures. Without any other mentions, plasma and serum are to be considered from human origin.

miRNA	Exosome source	Isolation technique	Exosome validation technique	Associated function	References
miR-21	(i) Cell supernatant (a) HCT-15, SW480, WiDR (b) CAFs, NOFs (c) Macrophages (ii) Serum (iii) Plasma (whole/mesenteric vs peripheral)	(i) UC (ii) UC/ExoQuick (iii) UC	(i) (a) WB : CsD81, (b) NTA/TEM/WB, (c) TEM/WB (ii) WB : CD81/none (iii) TEM/TEM + WB : Tsg101	Diagnosis biomarker, migration, invasion, liver metastasis, chemoresistance	[11, 99–105]

miR-155	(i) Plasma (whole/mesenteric vs peripheral) (ii) Cell supernatant (macrophages)	UC	(i) TEM/TEM + WB : Tsg101 (ii) NTA + TEM + WB	Migration, invasion	[104, 105]

miR-17∼92 and 25∼106b clusters	(i) Cell supernatant (SW480, SW620) (ii) Serum	(i) UC + OptiPrep (ii) qEV SEC/ExoQuick/UC	(i) TEM + WB (ii) EM + WB/-/TEM/TEM + WB	Diagnosis biomarker	[9, 23, 101, 106, 107]

miR-25-3p	(i) Cell supernatant (SW480, HCT116) (ii) Serum	UC	TEM + WB	Migration, angiogenesis, vascular permeability, pre-metastatic niches	[23]

miR-200 family	(i) Cell supernatant (a) SW640, SW480 (b) CCL27 (spheroid cultures) (ii) Plasma (mesenteric vs peripheral)	(i) (a) UC, (b) ExoQuick-TC (ii) UC	(i) (a) TEM, (b) None (ii) TEM, WB : Tsg101	EMT reversion marker, preventing permeation, associated with lower survival in exosomes	[98, 108–110]

miR-200b	Cell supernatant (SW480, HCT116)	UC	TEM + NTA	Proliferative activity	[98]

miR-1246	(i) Cell supernatant: (HCT116, HT29, SW480, Colo201, WiDR) (ii) Serum (iii) Plasma	UC	(i) WB : CD81/NTA/TEM (ii) WB : CD81 (iii) NTA + TEM + WB	Diagnosis biomarker, proliferation, migration, angiogenesis, pre-metastatic niches induction, TAM reprogramming	[11, 23, 102, 111]

mir-96 and mir-149	(i) Tissue (ii) Plasma	ExoCapTM + SG + FACS	TEM + WB : CD63	Tumor suppressor	[112, 113]

mir-486-5p	Plasma	Total exosome isolation kit	None	Tumor suppressor	[78, 114]

mir-6869-5p	Serum	Total exosome isolation kit	None	Prognostic biomarker, tumor suppressor	[78, 115]

mir-8073	Cell supernatant-HCT116	UC	None	Tumor suppressor	[116]

mir-193a	(i) Modified CT26 cells xenograft (ii) Serum	(i) UC + SG, pulldown (ii) exoEasy	NTA	Tumor suppressor	[92]

mir-10b	(i) Tissue (ii) Cell supernatant (HCT116)	UC + ExoQuick + exosomes precipitation solution	TEM-IG + WB	Oncogenic, CAF transformation	[117]

mir-142-3p	Cell supernatant (HCT-116, HT-29, SW480, MSCs)	UC	NTA + TEM-IG + WB	Induces stemness	[118]

mir-196b-5p	Serum	—	—	Induces stemness prognostic biomarker	[119]

mir-210	Cell supernatant (HCT-8)	Exosome precipitation solution	TEM	Induces EMT transition	[19]

EMT: epithelial-mesenchymal transition; FACS: fluorescence-activated cell sorting; MSCs: mesenchymal stem cells; OptiPrep: commercial density gradient medium; SG: sucrose gradient; TAM: tumor-associated macrophages; TEM/TEM-IG: electron microscopy (transmission/transmission coupled with immunogold labelling); UC: ultracentrifugation (may include differential centrifugation steps and eventual additional filtering); WB: western blot. Total exosome isolation kit (Invitrogen), ExoQuick (System Biosciences), exoEasy or exoRNeasy (Qiagen), qEV SEC (Izon), ExoCapTM (JSR), and exosome precipitation solution (Macherey-Nagel): commercial exosome purification solution or kits.

**Table 2:** Summary of exosomal miRNAs with a potential biomarker role in CRC or whose expression is associated with CRC diagnosis and progression. The main source of exosomes referred in the literature is indicated. Exosome isolation techniques employed in the diverse references are indicated with their respective exosome enrichment validation procedures. Without any other mentions, plasma and serum are to be considered from human origin.

miRNA	Exosome source	Isolation technique	Validation	Associated effect	References
mir-221	Cell supernatant (HT-15, SW480, WiDR)	UC	WB : CD81	Biomarker for tumor size, TNM stage, Dukes stage, lymph node metastasis, recurrence	[99, 135]

mir-215	(i) Cell supernatant (a) HT-15, SW480, WiDR (b) CAFs, NOFs	(i) UC (ii) UC	(i) WB : CD81/NTA + TEM + WB (ii) NTA + TEM + WB	Upregulation in CAF exosomes	[99, 103]

mir-23a	(i) Serum (ii) Cell supernatant (SW48, SW480, SW620, HCT116, HT29, RKO)	(i) UC (ii) UC	(i) TEM/WB (ii) TEM/WB	Diagnosis biomarker associated with liver metastasis recurrence	[9, 11]

mir-320a, -4476	(i) Tissue (ii) Serum	UC	TEM	Associated with liver metastasis recurrence	[9]

mir-125a	Plasma	ExoQuick	SEM/none	Early tumor stage biomarker	[27, 136]

mir-320c	Plasma	ExoQuick	SEM	Higher levels in CRC patients	[136]

mir-328	Plasma (mesenteric vs. peripheral)	UC	TEM + WB : Tsg101	Liver metastasis biomarker (better performing in mesenteric vein)	[105]

mir-4472-3p	Serum	ExoQuick	TEM + WB	Diagnosis and tumor recurrence biomarker	[137]

mir-6803-5p	Serum	Total exosome isolation kit	None	Diagnosis and prognostic biomarker, associated with stage and lymph node metastasis	[138]

mir-4644	Meta-analysis	—	—	GI cancer diagnosis biomarker	[102]

mir-7641	Cell supernatant (HT-15, SW480, WiDR)	UC + OptiPrep	TEM + WB : Alix, Tsg101	Diagnosis biomarker	[107]

mir-638	Serum	Total exosome isolation kit	None	Biomarker for TNM stages III-IV and liver metastasis	[77, 78]

mir-548c	(i) Serum (ii) Plasma (mesenteric vs peripheral)	(i) Total exosome isolation kit (ii) UC	(i) None (ii) TEM + WB : Tsg101	Diagnosis and prognostic biomarker, associated with faster relapse	[78, 105, 139]

mir-let-7a, -1229, -150, -223	(i) Cell supernatant (HCT116, HT29, RKO, SW48, SW480) (ii) Serum	UC	WB	Potential diagnosis biomarkers	[11]

GI cancers: gastrointestinal cancers; OptiPrep: commercial density gradient medium; TEM/SEM: electron microscopy (transmission/scanning); UC: ultracentrifugation (may include differential centrifugation steps and eventual additional filtering); WB: western blot. Total exosome isolation kit (Invitrogen), ExoQuick (System Biosciences), and qEV SEC (Izon): commercial exosome purification solution or kits.

## Abbreviations

CRC: Colorectal cancer
TME: Tumor microenvironment
mRNA: Messenger RNA
EV: Extracellular vesicle
MVB: Multivesicular body
MVP: Major vault protein
exomiR: Exosomal miRNA
NOF: Normal fibroblast
EPC: Endothelial progenitor cell
CAF: Cancer-associated fibroblast
TAM: Tumor-associated macrophage
EMT: Epithelial-mesenchymal transition
DAC: Decitabine
MET: Mesenchymal-epithelial transition
LEC: Lympho-endothelial cell
BEC: Blood endothelial cell
5-FU: 5-Fluorouracil
BM-MSC: Bone marrow mesenchymal stem cell
MHC: Molecular histocompatibility complex
HSP: Heat shock protein.
